# Daylight-PDT: everything under the sun

**DOI:** 10.1042/BST20200822

**Published:** 2022-04-06

**Authors:** Dana Beiki, Ian M. Eggleston, Charareh Pourzand

**Affiliations:** 1 Medicines Design, Department of Pharmacy and Pharmacology, University of Bath, Bath, U.K.; 2 Medicines Development, Centre for Therapeutic Innovation, University of Bath, Bath, U.K.

**Keywords:** ALA, cancer, daylight, PDT, skin, sun

## Abstract

5-Aminolevulinic acid-based photodynamic therapy (ALA-PDT) was first implemented over three decades ago and has since been mainly part of clinical practice for the management of pre-cancerous and cancerous skin lesions. Photodynamic therapy relies on the combination of a photosensitizer, light and oxygen to cause photo-oxidative damage of cellular components. 5-Aminolevulinic acid (ALA) is a natural precursor of the heme biosynthetic pathway, which when exogenously administered leads to the accumulation of the photoactivatable protoporphyrin IX. Although, effective and providing excellent cosmetic outcomes, its use has been restricted by the burning, stinging, and prickling sensation associated with treatment, as well as cutaneous adverse reactions that may be induced. Despite intense research in the realm of drug delivery, pain moderation, and light delivery, a novel protocol design using sunlight has led to some of the best results in terms of treatment response and patient satisfaction. Daylight PDT is the protocol of choice for the management of treatment of multiple or confluent actinic keratoses (AK) skin lesions. This review aims to revisit the photophysical, physicochemical and biological characteristics of ALA-PDT, and the underlying mechanisms resulting in daylight PDT efficiency and limitations.

## Introduction

**Figure 1.**
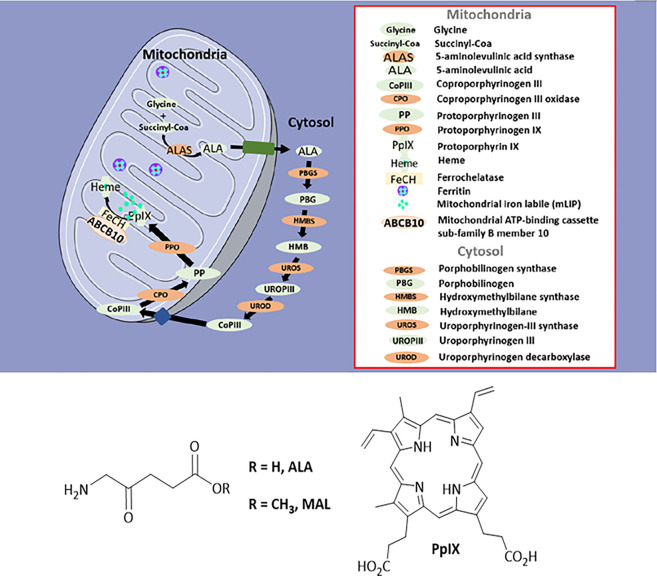
Schematic representation of the heme biosynthetic pathway. The chemical structures of ALA, its ester prodrug MAL, and the photosensitizer PpIX are shown beneath.

**Figure 2.**
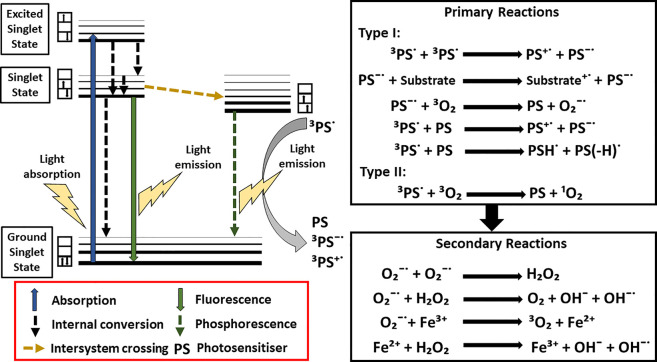
Representation of the photochemical reaction in PDT, as illustrated by the Jablonwski diagram (modified and adapted from Abrahamse and Hamblin [3]).

Photodynamic therapy (PDT) is a non-invasive treatment modality that uses the combination of a photosensitizer, light and oxygen to achieve the selective destruction of cells and tissues [1]. In the U.K., topical 5-aminolevulinic acid (ALA)-based PDT (ALA-PDT) has been widely accepted for actinic keratoses (AK), squamous cell carcinoma *in situ* (SCC) and basal cell carcinoma (BCC). ALA is a naturally occurring substrate of the heme biosynthetic pathway, which when added exogenously, results in the predominant accumulation of protoporphyrin IX (PpIX), a photoactivatable porphyrin. Exogenously added ALA bypasses the negative feedback control of 5-aminolevulinate synthase 1 (ALAS1), which ordinarily regulates heme production [2] (Figure 1). When a photosensitizer (e.g. PpIX) in the singlet ground state undergoes irradiation, the energy absorbed results in the production of an excited singlet electronic state. This may then undergo transition, through electron spin inversion to an excited triplet state [3] (Figure 2). This process is known as intersystem crossing. At this stage, the triplet state of the photosensitizer may undergo so-called type I or II reactions, resulting in the production of reactive oxygen species (e.g. singlet oxygen, ^1^O_2_) and the damage of biological components such as amino acids, unsaturated lipids, and DNA bases [3] (Figure 2). The photochemical process and subsequent reactions of PpIX in porphyrin-related PDT have been extensively reviewed [1,3,4]. The main cytotoxic agent of PpIX-based PDT is believed to be ^1^O_2_ [5]. Subsequently, these photochemical reactions may lead to cell death, PDT-induced vascular occlusion, and immune response [4–6]. Despite excellent treatment response and cosmetic outcome, the widespread adoption of topical ALA or its methyl-ester derivatives for PDT has been limited by the occurrence of adverse effects, such as pain, erythema, and oedema during the treatment [7,8]. In some cases, this has resulted in a significant number of patients undergoing ALA-PDT discontinuing the treatment [7]. The pain is experienced as a burning, stinging, and prickling sensation, which readily subsides once light treatment is stopped [9].

This review aims to reflect on the biological, physical, and chemical characteristics of ALA-PDT, supporting satisfactory treatment response and patient satisfaction in AK. More specifically the review will focus on the present indications for topical ALA-PDT related to management of AK. It will aim to assess the significance of the diffusion, distribution, and accumulation of the photosensitizer, and its interaction with different light sources, as well as distinguishing the ‘pros and cons’ of the emerging protocol of treatment, daylight PDT (dPDT) that is recommended for mild to moderate AK, with respect to treatment response and patient satisfaction.

### Treatment procedure

Levulan® Kerastick™ as a 20% w/v ALA solution is an FDA-approved formulation for the management of AK of the face, scalp, and upper extremities to be specifically used in combination with BLU-U blue light source (400–430 nm). In the U.K. ALA methyl-ester (MAL) is approved as Metvix®, which is a 16% w/w MAL cream used for the treatment of thin or non-hyperkeratotic and non-pigmented AK on the face and scalp. Recently, a 10% w/w ALA lecithin-based nanoemulsion was approved by the FDA as BF-200 ALA, and EMA as Ameluz®, for the site- and field-directed treatment of mild-to-moderate AK on the face and scalp [10,11]. Unlike FDA license that require the specification of both a prodrug and a specific source of light in combination, in the U.K. and Europe, the licenses for prodrugs Metvix and Ameluz allow their use with any narrowband or broadband light source (see [12,13]).

Conventionally, a 20% w/v ALA solution is applied on the skin, under occlusive dressing for a period of 14 to 18 h [14]. Similarly, 16% w/w MAL cream and Ameluz® gel are maintained under occlusive dressing, but for a shorter period of 3 h [14]. Light treatment varies based on the light source, with the use of BLU-U lamp (400–430 nm; 10 J/cm^2^) in the U.S.A., and either Aktilite 128 or a photodyn 750 lamp (630–635 nm; 37 J/cm^2^) in the U.K. and Europe. This protocol of treatment was first implemented in clinical practice and is now referred to as ‘conventional PDT’ (cPDT). Many variants of the original protocol have been investigated to improve the overall treatment and patient response. As such, dPDT was designed to reduce adverse reactions occurring during PDT treatment. dPDT consists of the application of Metvix® for 30 min, under occlusive dressing. The area is then cleaned, prior to irradiation with a ‘minimum’ weighted sunlight dose of 8 J/cm^2^ [15]. This light dose is referred to as the visible range of the spectral output weighted with the PpIX absorption spectrum [16]. However, 87% of PpIX photoactivation is attributed to the 380–495 nm waveband [16]. The detrimental effect of sunlight on ultraviolet (UV)-damaged skin is restricted by the use of an organic UV-blocking sunscreen [16]. In the U.K., sunlight treatment is practical between the months of March/April to October/September when the temperature is above 10°C, from 9:00 to 18:00 when the fluence rate reaches 130 W/m^2^ [17]. These limitations could be avoided with the use of an indoor daylight simulating lamp, providing a controlled environmental setting [18]. Many comparative studies have been undertaken to assess the efficacy of dPDT and cPDT in the management of AK [19–22] (Table 1). These results are conclusive, with an overall clearance ranging between 70% to 95% for dPDT and 74% to 80.6% for cPDT at the 10–12 months follow-up. dPDT exhibits comparable, if not superior clearance rate, with the advantage of being nearly painless. Similarly, the efficacy of dPDT using BF-200 ALA gel and MAL cream was compared in the treatment of grade I–III AK lesions [23]. BF-200 ALA was reported to exhibit significantly higher clearance rate for thin grade I AK lesions, however, its efficacy was comparable to MAL concerning thicker grade II–III AK lesions [23]. The same study reported that BF-200 ALA and MAL exhibited similar pain scores and cutaneous adverse effects [23].

**Table 1:** Non-exhaustive list of recent comparative Daylight PDT studies

Study	Indication	Preparation	Treatment protocol	Outcome
Wiegell et al. [24]	Face and scalp AKs (*n* = 29); Intra-individual/split-face study (cPDT vs dPDT)	Skin preparation; UVA + UVB sunscreen application; MAL cream (Metvix^®^) incubation under occlusive dressing for 3 h vs 30 min	Aktilite CL128 — Red LED light (632 nm; 37 J cm^−2^) vs 2.5 h daylight exposure	No significant difference in CR — 71% vs 79% LED treatment was more painful
Neittaanmä ki-Perttu et al. [23]	Face and scalp AKs grade I-III (*n* = 177); Intra-individual/split-face study (cPDT) (2 sessions for thicker grade AKs — 1 week apart)	25 min SPF20 sunscreen application; Skin preparation — curettage; MAL cream (Metvix^®^) vs BF-200 ALA (Ameluz^®^) gel incubation under occlusive dressing for 30 min	2 h daylight exposure	CR- 84·5% for BF200 ALA vs 74·2% for MAL; CR equal for thicker AK grade
Lacour et al. [20]	Face and scalp AKs (*n* = 100); Multi-centre RCT Intra-individual/splitface study (cPDT vs dPDT)	Skin preparation; UVA + UVB sunscreen application; MAL cream (Metvix^®^) incubation under occlusive dressing for 3 h vs 30 min	Aktilite CL128 — Red LED light (630 nm; 37 J cm^−2^) vs 2 h daylight exposure	No significant difference in CR — 70% vs 74% d-PDT nearly painless (11-point numeric rating scale; 4.4 vs 0.7)
Cantisani et al. [21]	Face and scalp, nose, trunk, and extremities AKs (*n* = 646); Retrospective study (cPDT vs dPDT)	MAL cream incubation under occlusive dressing for 3 h vs 30 min	Aktilite CL128 — Red LED light (630 nm; 37 J cm^−2^) vs 2 h daylight exposure	CR — 74.4% vs 95% dPDT CR was best for AK size ≥ 0.6 mm
Sotiriou et al. [22]	Face and scalp AKs (*n* = 26); Intra-individual/split-face study (cPDT vs dPDT) (two sessions — 1 week apart)	Skin preparation; SPF20 sunscreen application; MAL cream incubation under occlusive dressing for 3 h vs 30 min	Aktilite CL128 — Red LED light (630 nm; 37 J cm^−2^) vs 2 h daylight exposure	No significant difference in safety and efficacy of cPDT vs dPDT; Higher local adverse events with cPDT but equal preventive potential with dPDT

Abbreviations: AKs, Actinic keratosis; RCT, Randomized controlled trial; cPDT, conventional PDT; dPDT, daylight PDT; CR, Clearance rate or clinical response.

### Topical photosensitizer delivery

The clinical success of topical PDT relies partly on the physicochemical properties of the photosensitizer. These determinants account for the specific drug diffusion, distribution, and accumulation in the tissue, as well as ease of administration and stability. An important limitation to topically applied ALA is its permeability through the stratum corneum, and therefore its bioavailability to subsequent epidermal and dermal layers [25]. The stratum corneum is often illustrated as the ‘brick and mortar’ model, where anucleated keratinocytes are embedded in lipid bilayers, functioning as a natural barrier. According to Fick's first law, molecular diffusion is passive and thermodynamically stable, as the drug is diffusing from high to low concentrations until equilibrium [26]. In this way, there is a requirement for a favorable partition coefficient and photosensitizer molecular weight for the permeation to the stratum corneum, and more generally, transdermal drug delivery [27].

#### Diffusion

ALA has a low molecular weight (131.12 g/mol) and due to its hydrophilic nature, it exhibits a low partition coefficient (log P = −1.5) [28]. These chemical characteristics result in a non-homogenous permeation, restricted to depths of ∼1 mm [28], limiting its clinical application to superficial lesions [29]. Although many permeability-enhancing approaches have been investigated, the chemical modification of the carboxylic acid group to produce an ester prodrug has also been widely implemented as a solution.

Esterification of the carboxyl group renders MAL more lipophilic than ALA, inducing PpIX fluorescence superficially and within the area of application, whereas ALA induced-PpIX occurs away from treated skin areas [30]. In this way, the diffusion of ALA is more extensive than MAL, with prolonged ALA application resulting in systemic photosensitization [30]. However, this systemic effect is not observed with MAL [31]. According to Fick's first law of mass flow, ALA-induced PpIX fluorescence should decrease in relation to increasing depth. However, this linear relation between PpIX fluorescence and depth only exists in the epidermis [32]. Despite fibroblasts being capable of accumulating PpIX, their spatial arrangements and cellular density partly explain the dissimilarity in PpIX accumulation when compared with keratinocytes in the epidermis [26]. Upon topical application of ALA, dermal cells and connective tissue exhibit respectively, ∼45% and 10%, of the porphyrin fluorescence intensity present in the epidermis [33]. Similarly, ALA application has been reported to display an enhanced porphyrin accumulation in sebaceous glands and hair follicles [29,31], but represents just a small fraction of the porphyrin fluorescence present in the epidermis [31].

Many studies have tried to map PpIX distribution within the skin, but their findings have been mixed and conflicting. Pro-drug-induced PpIX distribution matches the type of lesion studied, the technique utilized, and the skin type and location investigated. However, it can be hypothesized that dPDT procedure is restricted to the diffusion profile of MAL, and therefore, predominantly to the treatment of lesions of the epidermis. This diffusion profile dissimilarity was suggested to correlate with less pain. As such, Wiegell et al. [24] reported that ALA-PDT was more painful than MAL–PDT in healthy skin. Comparably, MAL–PDT was later confirmed to be less painful than ALA-PDT in the treatment of multiple AKs of the scalp [7]. ALA is thought to sensitize peripheral nerve endings, whereas MAL neural stimulation is thought to be minimal [34]. It can therefore be suggested that the continuous photoactivation of PpIX after a 30 min application of MAL, leads to a more pronounced accumulation in the most superficial layers of the skin, and therefore causes, minimal neural sensitization at the dermal-epidermal junction [35,36]. This can also suggest a reason why conventional PDT efficacy is superior for deep-rooted skin lesions, notably in thicker AK, BCC and Bowen's disease, when compared with dPDT [17]. In general pain with widespread AK is an issue for cPDT which justifies the use of dPDT for AK. However for conditions such as small single BCC lesions, the pain has not been reported as an issue for cPDT and in fact, dPDT is not licensed for this indication [37,38]. The latter is because dPDT works best for mild to moderate AK and is not effective for thicker lesions.

#### Distribution

Originally, the more lipophilic nature of MAL when compared with ALA was thought to improve its transdermal delivery. However, animal studies demonstrated that MAL application did not result in higher PpIX production when compared with ALA, but rather preferential PpIX accumulation in AK lesions as compared with adjacent healthy skin [39]. Similarly, Van den Akker et al. [40] confirmed through tape stripping of normal skin that ALA permeation was significantly higher than MAL. A comparative *in vivo* study of ALA-induced PpIX in skin tumors between altered adjacent skin and healthy adjacent skin in UVB-treated mice established that the fluorescence increase was significantly higher in tumor and altered tissue than in normal tissue [41]. At the 10 h period post-topical application of ALA solution, the mean fluorescence in tumor tissue was found to be 1.4 times higher than altered tissue, which was respectively 4 times higher than normal tissue [41]. This selective accumulation was even more significant with topical MAL application, where the selective porphyrin accumulation in neoplastic lesions was 10-fold the fluorescence in normal tissue [42].

PpIX intensity is indicated as the most important predictor of treatment response [43]. Although no clear correlation exists between fluorescence and treatment response, Fink-Puches et al. [44] have reported a statistically significant correlation between moderate to very strong fluorescence intensity and clearance rate. Therefore, PDT treatment may require a PpIX concentration threshold in order to be effective. In addition, it has been demonstrated that the mode of cell death and treatment efficacy were dependent on the subcellular localization of the photosensitizer [45]. Generally, the subcellular location of the photosensitizer is determined by the cell type, photosensitizer chemistry as well as its initial concentration [45]. In clinics, the mode of cell death is hard to define, due to post-synthetic migration of PpIX [46] and its dependence on the ALA treatment period [47]. In practice, several modes of cell death are thought to be triggered simultaneously [46]. The effect of PpIX subcellular localization on the mechanism underlying its cytotoxicity is partly due to the short-lived ^1^O_2_, that only causes oxidation of biological compounds present in its vicinity [3]. PpIX is first synthesized in the mitochondria (see Figure 1), but diffuses out to other biological compartments, such as the endoplasmic reticulum (ER) and lysosomes due to its hydrophobic nature [45]. Two-photon excitation fluorescence microscopy has established that ALA-incubated DHL cells exhibited PpIX fluorescence mainly in the ER, and at lower levels in the mitochondria, with residual fluorescence present in the lysosomes [45]. Wilson et al. [48] reported that ALA-incubated RIF cells exhibited significantly more clonogenic survival after a long ALA incubation (5 h) when compared with a shorter 1 h ALA incubation period. The authors reported that enhanced PpIX post-synthetic migration was taking place with longer ALA incubation periods (5 h), suggesting the mitochondria as the most sensitive subcellular target [48]. Generally, the photo-oxidative damage of the ER and mitochondria results in apoptosis [46], whereas the photo-oxidative damage of lysosomes result in less photokilling via non-apoptotic pathways, involving autophagy [49]. Additionally, incubation with high ALA concentrations has been shown to cause necrosis [50]. In this way, the design of the dPDT treatment would predominantly favor apoptosis. In the context of dPDT, PpIX accumulation is occurring during irradiation, inflicting most of the photo-oxidative burden on the mitochondria. Accordingly, dPDT is associated with less adverse skin reactions than cPDT. This was demonstrated in a phase III RCT, where dPDT was shown to cause less adverse reactions, such as erythema, scab, skin burning sensation, when compared with cPDT [20]. McLellan et al. [51] reported 82% of patients were satisfied with the dPDT procedure.

One way to increase the effectiveness of PpIX-mediated photokilling independent of ALA precursor concentration for both cPDT and dPDT would be to design organelle-targeted PpIX photosentizers based on strategies involving organelle-homing peptides [52–54].

#### Accumulation

**Figure 3.**
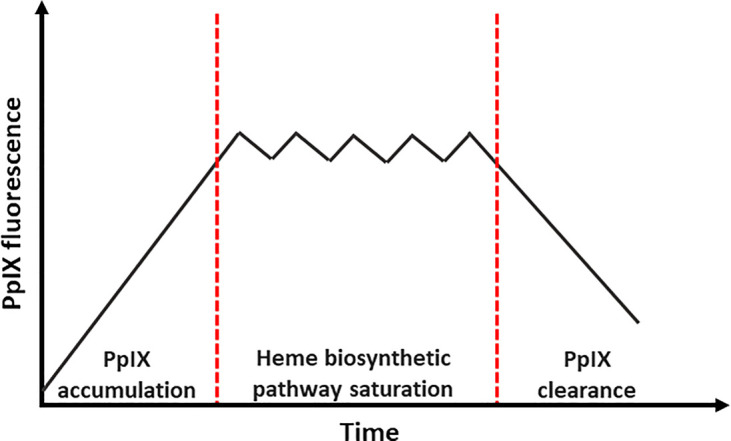
Representation of the three-stage pro-drug induced PpIX accumulation in the skin.

Mathematical modeling of topical ALA-induced PpIX formation in healthy skin has been shown to successfully fit experimental data [31]. This encompassed a three-compartment model, with first the time-dependent diffusion of ALA or the formation of an extracellular ALA sink, then the intracellular ALA uptake and conversion to PpIX, followed by its clearance to non-fluorescing heme and bilirubin [31]. This model reported that only a fraction of ALA passing the stratum corneum (2.5–3.5%) would be converted to PpIX [31]. Although the authors suggested the need to further refine the model, the three-compartment model clearly fitted the three-stage PpIX fluorescence time course observed upon topical ALA and MAL application (Figure 3). In the initial stage, the rate of PpIX accumulation decreases until equilibrium is reached. This equilibrium represents an intermediate stage where the PpIX accumulation rate flattens due to saturation of the heme biosynthetic pathway. The length of this stage depends solely on the application period of ALA and its esters [10,55]. The last stage represents the falling of the rate of PpIX accumulation or the rate of PpIX clearance, either through metabolism into heme or bilirubin, or extracellular diffusion [30]. In a fluorokinetic study, the notion of the extracellular ALA sink was supported as no statistical difference between a 1 h and 4 h ALA application period up to 7 h after ALA application was established [56]. The same authors later found that topical application of ALA and MAL for 4 h did not result in any significant difference in PpIX fluorescence up to 7 h after their application [10]. Accordingly, it was only at 24 h post-prodrug application that a significant difference in PpIX fluorescence was observed [10]. This confirmed prior findings by Juzenas et al. [57], on the enhanced MAL-induced PpIX clearance over ALA prodrug. This suggests that the design of the dPDT protocol allows the photoactivation of PpIX in its accumulation stage, suggesting a reduced post-treatment photosensitization due to lower time-dependent diffusion of MAL. Moreover, the continuous photoactivation of PpIX correlates with PpIX production, predicting efficient subcellular targeting, notably mitochondrial, and reduced accumulation in the skin [35].

### Light delivery — absorption spectrum, photobleaching and fluence rate

PpIX displays an absorption spectrum with a maximum at 400 nm referred to as the Soret peak, and four regional maxima between 500 and 635 nm referred to collectively as Q-bands [58]. Generally, wavelength ranges between ∼400 and 420 nm are referred to as blue, whereas wavelength ranges between ∼570 and 700 nm are referred to as red. Light treatment in PDT has attempted to exploit both of these absorption regions which provide distinct depth-dependent photochemical characteristics [59,60]. Ash et al. [61] modeled the penetrative depth of light of different wavelengths and established that wavelengths between 350 to 400 nm reached depths of 750 µm to 1000 µm, whereas wavelengths between 550 to 650 nm reached depths of 3000 µm up to 5000 µm. Furthermore, non-uniform tumoral tissue structure was shown to result in different light penetration and PpIX distribution [62]. Additionally, PpIX photoactivation is wavelength-dependent. An *in vitro* study led by Buchczyk et al. [63] determined that similar PDT doses with UVA and green light caused 40- and 10-fold more photokilling than red light in ALA-incubated skin fibroblasts. It is also noteworthy that other chromophores absorb incoming photons, further complicating the wavelength-PpIX photoactivation relationship. In this way, relative photoactivation is not solely based on the photophysical properties of PpIX alone. This notion is even more important for broad spectrum light sources, such as the natural sunlight. Furthermore, combining light wavelengths could have a synergistic effect in the photoactivation of PpIX and resulting ROS production. This was observed with dual-wavelength irradiation combining 405 nm and 505 nm, where the relative tumor volume in mice was significantly reduced after 20 days when compared with 405 nm alone [64]. In this way, it can be suggested that the use of broad-spectrum light sources such as sunlight reduces the overall treatment time, when compared with a narrower waveband. As a result, when comparing narrowband light with sunlight irradiation, sunlight can most efficiently photoactivate PpIX within the same period. This could therefore explain how dPDT effective light doses above 8 J/cm^2^ result in similar or superior treatment efficacy when compared with cPDT [14].

In the process of photoactivation, the photosensitizer undergoes photochemical degradation, which is referred to as photobleaching. The photobleaching rate is dependent on three factors, PpIX and oxygen concentrations, as well as wavelength delivery [65,66]. Mikolajewska et al. [9] established that with fluence rates of blue/violet (405 nm) and red light (632 nm) of 8 mW/cm^2^ and 100 mW/cm^2^, respectively, a comparable fraction of PpIX photobleaching was achieved. In theory, comparable clearance rate could be achieved within shorter treatment periods with blue light when compared with red light, if delivered at similar fluence rates. Indeed, Moan and colleagues found that the yield of PpIX photobleaching per incident photon at 420 nm was 15- to 20-fold higher when compared with 632 nm [67,68]. Correspondingly, the majority of PpIX photobleaching per incident photon is attributed to the 380–495 nm waveband [13]. In this regard, natural sunlight displays an efficient light dose to PpIX photoactivation ratio, inflicting maximum photodamage at minimal light dose [66].

Fluence rate is also considered as a key factor in pain moderation and patient satisfaction. It has been demonstrated that treatment efficiency is maintained at lower fluence rates, at the expense of longer treatment periods [69,70]. However, the mean pain scores were found to be significantly less for fluence rates below 50 mW/cm^2^ [16,71]. The minimal fluence rate at which dPDT is effective falls under this threshold, further preventing high-level activation of PpIX and treatment-induced pain. It is also noteworthy to mention that fluence rate is believed to vary at different wavelength and depth, due to the structural non-uniformity of the skin [58]. In this regard, the dynamics between prodrug accumulation and subsequent light photoactivation becomes more complicated for thicker and deeper skin lesions.

## Conclusion

dPDT is an established cost-effective alternative to cPDT for the management of mild to moderate AK lesions, displaying improved patient compliance and adherence, effective management of healthcare services, and relatively low variability of outcomes. Treatment effectiveness is maintained during cloudy days, and it has even been reported to result in less pain [72]. Similarly, treatment could be undertaken in a greenhouse or under artificial light [15]. The dPDT protocol fully exploits the photophysical, physicochemical and biological properties of the ALA-based prodrug, light source, and skin, to provide effective treatment response and patient satisfaction. The protocol design allows the efficient continuous photoactivation of superficial tumoral PpIX accumulation, via the shortening of prodrug administration. This causes the effective diffusion, distribution and the selective accumulation of prodrug- induced PpIX, suggesting enhanced selective photodamage. As a result, dPDT provides improved patient adherence and satisfactory clearance rate when compared with cPDT. The specific design of the dPDT protocol is also responsible for its limitations, restricting its application to thin and superficial lesions of the skin. However, compared with cPDT, dPDT treatment is time- and cost-effective, using less significant resources. Sunlight results in superior PpIX photoactivation, permitting the delivery of an effective light dose within practical timeframes.

## Perspectives

- dPDT was designed for the management of large areas of the skin lesions, resulting in minimal pain and skin reactions.
- Comparative studies for the management of thin AK skin lesions, have suggested that dPDT treatment outcome is either comparable or superior to cPDT.
- Protocol design alterations, such as prodrug administration and daylight exposure could further improve treatment outcome and patient satisfaction with thicker and deep-rooted AK skin lesions.

## Open Access

Open access for this article was enabled by the participation of University of Bath in an all-inclusive *Read & Publish* agreement with Portland Press and the Biochemical Society under a transformative agreement with JISC.

## Abbreviations

^1^O_2_: singlet oxygen
AK: actinic keratoses
ALA: 5-Aminolevulinic acid
ALA-PDT: 5-Aminolevulinic acid-based photodynamic therapy
ALAS1: 5-aminolevulinate synthase 1
BCC: basal cell carcinoma
cPDT: conventional photodynamic therapy
dPDT: daylight photodynamic therapy
ER: endoplasmic reticulum
MAL: ALA methyl-ester
PDT: Photodynamic therapy
PpIX: protoporphyrin IX
SCC: squamous cell carcinoma
UV: Ultraviolet
UVA: ultraviolet A
UVB: ultraviolet B
